# Coordination Polymers Based on Phthalic Acid and Aminopyrazine Ligands: On the Importance of N–H···π Interactions  

**DOI:** 10.3390/polym10020182

**Published:** 2018-02-13

**Authors:** Anowar Hossain, Saikat Kumar Seth, Antonio Bauzá, Subrata Mukhopadhyay, Antonio Frontera

**Affiliations:** 1 Department of Chemistry, Jadavpur University, Kolkata 700032, India; anowar.hossain.chem@gmail.com (A.H.); smukhopadhyay@chemistry.jdvu.ac.in (S.M.); 2 Department of Physics, Jadavpur University, Kolkata 700032, India; 3 Department of Chemistry, Universitat de les Illes Balears, Crta. de Valldemossa km 7.5, 07122 Palma de Mallorca (Baleares), Spain; antonio.bauza@uib.es

**Keywords:** Co(II) and Cu(II) polymers, phthalic acid, N–H···π interaction, DFT calculations

## Abstract

Two new Co(II) and Cu(II) coordination polymers, {Co(HL_1_)_2_(μ-L_2_)(H_2_O)_2_}*_n_* (**1**) and {[Cu(HL_1_)_2_(μ-L_2_)H_2_O]·H_2_O}*_n_* (**2**) (H_2_L_1_ = Phthalic acid and L_2_ = 2-aminopyrazine), have been synthesized by slow evaporation of solvent and characterized by IR spectroscopic, elemental, single-crystal X-ray diffraction and thermal analysis. X-ray results indicate that in both the polymers, phthalate acts as a monodentate ligand and the aminopyrazine ligand is responsible for the formation of the infinite one-dimensional chain structure. The solid-state structures are stabilized through hydrogen bonds and N‒H···π interactions by generating two-dimensional layered structures. Finally, the non-covalent interactions have been studied energetically and using Bader’s theory of atoms in molecules by means of Density Functional Theory (DFT) calculations. The influence of the metal coordination on the strength of the interaction has been studied using molecular electrostatic potential surface calculations.

## 1. Introduction

Coordination polymers include a large family of compounds that are formed by central metal ions linked to a variety of ligands by coordination bonds. They have attracted much attention over the last decade due to their fascinating structures and, more importantly, their potential applications in catalysis, gas storage, luminescence, and sensing, among others [1,2,3,4]. Their final solid state structure is driven by a self-assembly process where, depending on the selected building blocks, gives rise to mono-, bi-, or three-dimensional structures. The building blocks of the coordination polymers determine their physical and chemical properties [5,6,7,8].

Interestingly, one- or two-dimensional coordination polymers can organize their three-dimensional architecture by means of supramolecular interactions, frequently hydrogen bonding or less directional van der Waals forces. However, the formation of self-assembled large supramolecular aggregates is also governed by supramolecular interactions involving aromatic rings. To this respect, C–H···π [9], π···stacking [10,11,12], cation···π [13], anion···π [14,15,16,17], and lone pair···π [18,19,20] interactions are prominent binding forces [21] that have been used successfully used to build solid-state networks [22]. The proper understanding of these forces is important not only for rationalizing the existing solid state architectures of these compounds but also to be able to design and predict new supramolecular entities based on mono-dimensional coordination polymers.

**Scheme 1:**
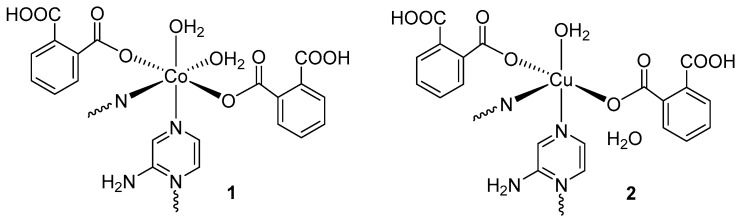
Chemical diagrams of the compounds **1** and **2**.

Keeping this in mind, two new Co(II) and Cu(II) coordination polymers, {Co(HL_1_)_2_(μ-L_2_)(H_2_O)_2_}*_n_* (**1**) and {[Cu(HL_1_)_2_(μ-L_2_)H_2_O]·H_2_O}*_n_* (**2**) (H_2_L_1_ = Phthalic acid and L_2_ = 2-aminopyrazine), have been synthesized and X-ray characterized (see Scheme 1). X-ray results indicate that both polymers exhibit one-dimensional chain structure. The solid-state structures are stabilized through hydrogen bonds and N‒H···π interactions. The short N‒H···π non-covalent interactions have been analyzed both energetically and using Bader’s theory of “atoms in molecules” by means of Density Functional Theory (DFT) calculations using several theoretical models of the polymers. In particular, we have studied the effect of the metal coordination on the strength of the N‒H···π interaction.

## 2. Materials and Methods 

### 2.1. Materials

The reagents were purchased from commercial sources and used without further purification. Freshly boiled, doubly distilled water was used throughout the synthesis of the compounds.

### 2.2. Instruments

IR spectra (KBr disk) were performed on a Perkin-Elmer RXI FT-IR spectrophotometer (Perkin Elmer Inc., Waltham, MA, USA). Elemental analyses were carried out on a Perkin-Elmer 240C elemental analyzer. Thermogravimetric analysis (TGA) data were collected under nitrogen atmosphere in the temperature range of 21 °C to 850 °C at a heating rate of 10 °C/min with an DTG 60H, Shimadzhu thermo-analyzer (Shimadzhu Coorporation, Kyoto, Japan). X-ray diffraction measurements were conducted on a Bruker APEX-II CCD (Bruker GmbH, Mannheim, Germany).

### 2.3. Preparations

#### 2.3.1. General Procedure

Compounds **1** and **2** were prepared by reacting stoichiometric amounts of metals and ligands in aqueous solution. For **1**, 1 mmol Cobalt(II) nitrate hexahydrate was mixed with 2 mmol of phthalic acid and 2 mmol of 2-aminopyrazine in aqueous medium, stirred for 2 h at 60 °C. The resulting solution was allowed to cool to room temperature, filtered and kept undisturbed for crystallization. Suitable single crystals for X-ray analysis were obtained after a few weeks. For **2**, 1 mmol Copper(II) nitrate trihydrate was reacted with 2 mmol phthalic acid and 2 mmol 2-aminopyrazine and followed the same procedure as in **1**. The crystals were collected by filtration, washed with cold water, and dried in the air.

#### 2.3.2. {Co(HL_1_)_2_(μ-L_2_)(H_2_O)_2_}*_n_* (**1**)

Yield: 0.340 g (65.5%). Anal. Calcd. for C_20_H_18_CoN_3_O_10_ (*M_W_* = 519.3): C, 46.26; H, 3.49; N, 8.09%. Found: C, 46.21; H, 3.44; N, 8.13%. FT–IR (cm^−1^): 3750(s), 3464(s), 3305(b), 3207(b), 2796(b), 2487(b), 1898(b), 1690(s), 1633(s), 1590(s), 1533(s), 1490(s), 1448(s), 1411(s), 1261(s), 1211(s), 1146(s), 1076(s), 1032(s), 954(s), 882(s), 818(s), 767(s), 689(s), 646(s), 589(s), 550(s), 524(s), 439(s).

#### 2.3.3. {[Cu(HL_1_)_2_(μ-L_2_)H_2_O]·H_2_O}*_n_* (**2**)

Yield: 0.318 g (60.5%). Anal. Calcd. for C_20_H_19_CuN_3_O_10_ (*M_W_* = 524.9): C, 45.76; H, 3.65; N, 8.00%. Found: C, 45.70; H, 3.62; N, 8.07%. FT–IR (cm^−1^): 3473(b), 3327(b), 2930(s), 2618(b), 2492(b), 1741(s), 1699(s), 1623(s), 1567(s), 1519(s), 1491(s), 1442(s), 1394(s), 1262(s), 1206(s), 1144(s), 1068(s), 1012(s), 942(b), 886(s), 858(s), 810(s), 768(s), 740(s), 699(s), 650(s), 587(s), 559(s), 517(s).

### 2.4. X-ray Details

Single crystal X-ray diffraction intensity data of the title compounds were collected at 100(2)K and 293(2)K for **1** and **2** respectively, using Bruker APEX-II CCD diffractometer equipped with graphite monochromated MoKα radiation (λ = 0.71073 Å). Data reduction was carried out using the program Bruker SAINT [23] and an empirical absorption correction was applied based on multi-scan method. The structures of the title compounds were solved by direct method and refined by the full-matrix least-square technique on F^2^ with by using the programs SHELXS-14 and SHELXL-18 [24], respectively. All H-atoms were located from difference Fourier map. All calculations were carried out using WinGX system Ver-1.64 [25] and PLATON [26]. To analyze the non-covalent interactions, (.lps) and (.sup) files were generated by using PLATON. A summary of crystal data and relevant refinement parameters are given in Table 1. CCDC 1816232-1816233 contain the supplementary crystallographic data for this paper.

### 2.5. Theoretical Methods

The energies of the H-bonding interactions using minimalistic models of the polymers were computed using the BP86-D3/def2-TZVP level of theory by means of the program TURBOMOLE version 7.0 (TURBOMOLE GmbH, Karlsruhe, Germany) [27] and the crystallographic coordinates. The binding energies were computed applying the correction for the BSSE (basis set superposition error) by using counterpoise technique developed by the Boys–Bernardi [28]. The Bader’s “Atoms in molecules” (AIM) theory [29] was employed to analyse the interactions studied herein by means of the AIMall program [30].

## 3. Results and Discussion

### 3.1. Structural Description of Compounds ***1*** and ***2***

**Figure 1:**
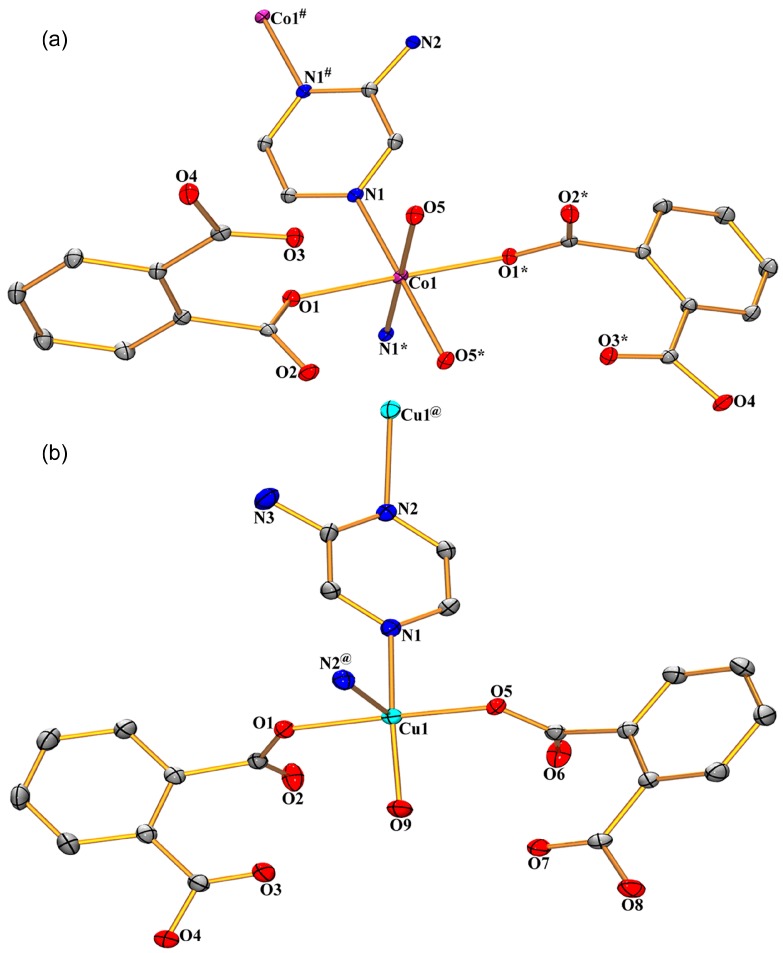
Central atom environments (Oak Ridge Thermal Ellipsoid Plot (ORTEP) view) and atom numbering schemes of the title polymers (**a**) **1** and (**b**) **2**. The 2-aminopyrazine moiety is disordered over an inversion centre in **1** where the amino group have partial site occupancy factor of 0.5. In **2**, the amino group of the 2-aminopyrazine moiety have 0.7 and 0.3 occupancies. Only one amino group with fractional site occupancies have been shown for both compounds. Solvent water molecule in **2** and hydrogen atoms for both structures are omitted for clarity. Thermal ellipsoids are drawn at 30% probability.

Crystal structure analysis reveals that compound **1** is a one-dimensional metal–organic coordination polymer constructed from Co(II) ion, phthalic acid, and 2-aminopyrazine. Two nitrogen atoms from two mono-aminopyrazine ligands and two oxygen atoms from two phthalate ligands and two solvent water oxygen atoms surround the crystallographically independent Co(II) ion, in a distorted octahedral geometry {CoN_2_O_4_} (Figure 1a). Co(II) ion is in inversion center (−*x* + 1, *y*, −*z* + 1/2) whereas the half of the mono-aminopyrazine molecule is generated through a symmetry operation of (−*x* + 1, −*y*, −*z*). In the crystal structure, the crystallographically unique amino group (in general position) was refined with a site occupancy factor of 0.5. The Co-N bond length is 2.171(2) Å, and the Co-O bond lengths fall in the range 2.048(2)–2.125(2) Å, which are well in agreement with those reported in other Co(II) complexes with N,O-mixed ligands [31]. The bond lengths and angles surrounding the metal center are included in Table S1.

**Figure 2:**
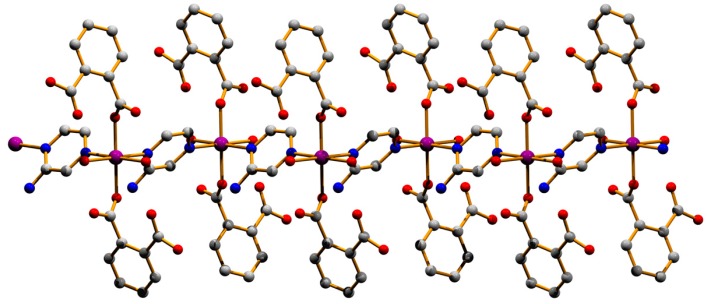
Propagation of one-dimensional polymer along (001) direction in **1**. One amino group of the aminopyrazine moiety with partial occupancy factor has been used to draw the figure.

**Figure 3:**
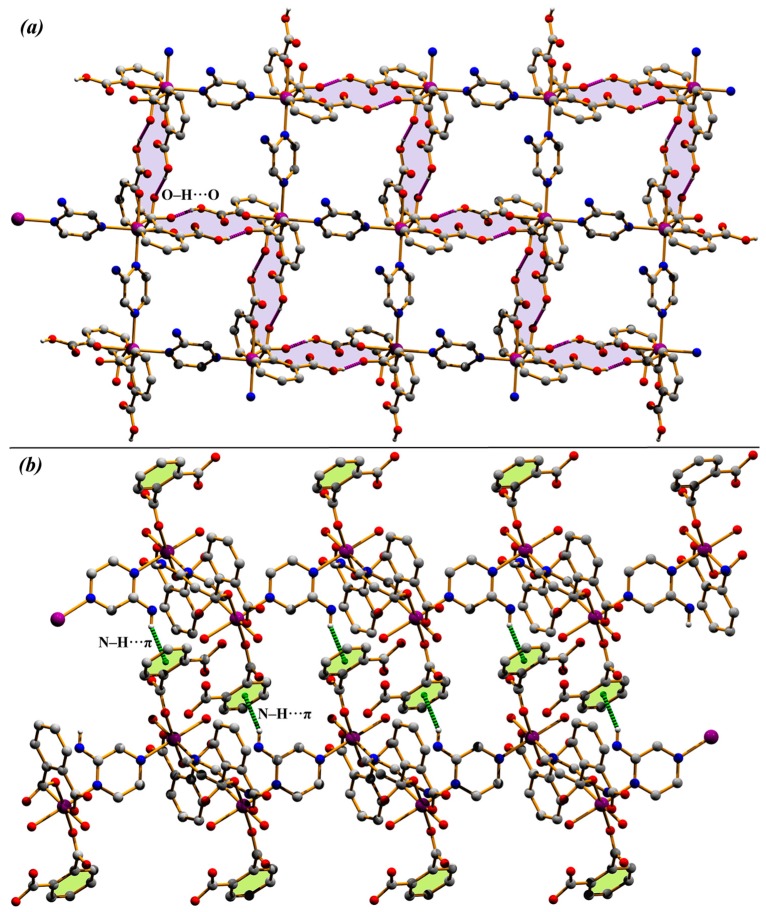
(**a**) Two-dimensional network in (011) plane through O‒H···O hydrogen bonding interaction. (**b**) Layered framework in (011) plane through N‒H···π interaction in **1**. One amino group of the aminopyrazine moiety with partial occupancy factor has been used to draw the figures.

The solid-state structure of **1** includes a combination of O‒H···O, C‒H···O hydrogen bonds and N‒H···π interactions (Table 2). In the first sub-structure, the molecules are propagating along (001) direction through the coordination-bonding mode thus generating the one-dimensional coordination polymer (see Figure 2). These one-dimensional polymeric chains are interconnected through self-complementary hydrogen bonds and generates a two-dimensional assembly. The carboxylate oxygen atom O(4) in the molecule at (*x*, *y*, *z*) acts as donor to the carboxylate carbonyl atom O(2) in the molecule at (*x*, 1 − *y*, −1/2 + *z*); thus forming a R_2_^2^(22) ring motif (see Figure 3a). This ring motif binds the parallel polymeric chain and leads the molecules to form a two-dimensional assembly in (011) plane (Figure 3a). In another sub-structure, the amino nitrogen atom N(2) is oriented toward the π-face of the aryl ring of phthalate molecule at (−1/2 + *x*, 1/2 − *y*, −1/2 + *z*); thus interconnecting two parallel polymeric network into a layered assembly in (011) plane (Figure 3b). 

**Figure 4:**
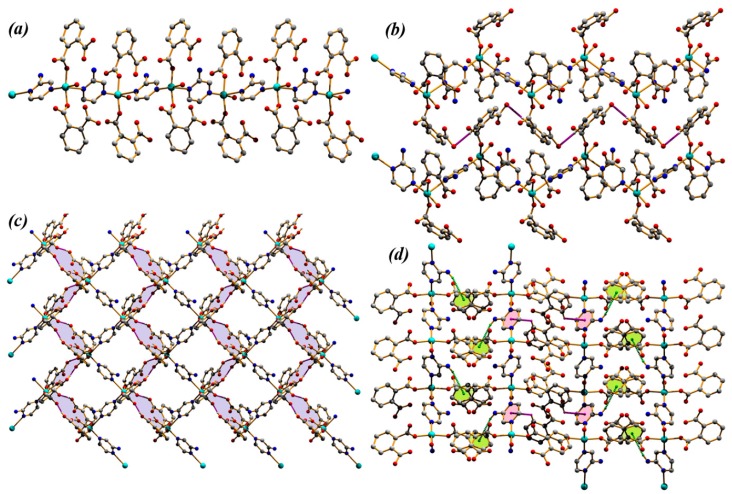
(**a**) Polymeric chain structure in **2**; (**b**) Interconnection of the parallel polymeric chains; (**c**) Formation of two-dimensional framework where R_2_^2^(22) acts as building block of the structure; (**d**) Layered network in (011) plane where the green dotted lines represent N‒H···π interaction, whereas the pink dotted lines are indicating C‒H···π interactions in **2**. In the solid-state structures, the amino group from 2-aminopyrazine moiety that have higher occupancy factor is taken into consideration to draw the figures. Disordered counterpart of amino group with small occupancy factor is omitted for clarity.

The asymmetric unit of the solid-state structure of **2** is depicted in Figure 1b with atom numbering scheme, where the Cu(II) ion exhibits a five-coordinate elongated (4 + 1) square pyramidal geometry with the phthalate anion as primary ligand and with 2-amino pyrazine as bridging ligand. The amino group of the 2-aminopyrazine moiety is also affected by disorder in the structure and have mirror disordered parts with partial site occupancy factors of 0.7 and 0.3, respectively. Two O atoms of two phthalate anions, one water O atom and one N atom of the bridging ligand, form the base of the square-pyramid. The apex of the pyramid is occupied by the N atom of the pyrazine ligand which links the [Cu(L_1_)_2_(L_2_)(H_2_O)] unit into infinite polymeric chain running along (010) direction (Figure 1b and Figure 4a). One solvent water oxygen atom, which is not bonded with the polymeric unit, is not shown in Figure 1b. The Cu–O lengths [Cu(1)–O(1) = 1.949(3), Cu(1)–O(5) = 1.931(3), and Cu(1)–O(9) = 1.957(3) Å] are in good agreement with those found in related structures (1.923–2.049 Å) [32]. The Cu–N lengths [Cu(1)–N(1) = 2.023(3), Cu(1)–N(2) = 2.513(3) Å] are comparable with the ones found in the other Cu(II) imine complexes [33]. The geometrical parameters surrounding the metal ion are included in Table S2. In the polymeric chain structure, two adjacent Cu(II) pyramids are lying in the same direction and thus forming a linear chain structure (Figure 4b). In the solid-state, covalently bonded polymeric structure exhibits non-covalent interactions such as hydrogen bonds, C–H···π and N–H···π interactions (Table 2). The aryl ring carbon atom C(3) of the phthalate moiety in the molecule at (*x*, *y*, *z*) acts as donor to the carboxylate oxygen atom O(4) of the partner molecule at (1 − *x*, −1/2 + *y*, 3/2 − *z*); thus interlinks the parallel polymeric chains propagating (010) direction into a layered assembly in (110) plane (Figure 4b). In another substructure, two carboxylate oxygen atoms O(4) and O(8) of phthalate molecules acting as donors to the oxygen atoms O(2) and O(6) of the metal-coordinated carboxylate groups, respectively. The interactions in between the carboxylate oxygen atoms in the molecule at (*x*, *y*, *z*) and (1/2 − *x*, 1/2 + *y*, *z*) generates a R_2_^2^(22) dimeric ring, repetition of which zigzag polymeric chain are interlinked and thus a two-dimensional network is generated in the (110) plane (Figure 4c and Figure S3). Again parallel polymeric chains along (010) are interconnected through N–H···π interaction and strengthen the network (Figure 4d). The amino nitrogen atom at (*x*, *y*, *z*) acts as donor to the π-cloud of the aryl ring of the phthalate moiety in the molecule at (1/2 + *x*, *y*, 3/2 − *z*). Interestingly, two nearby parallel chains are interconnected due to the self-complementary nature of the phthalate ring carbon atom and the π-cloud of the pyrazine ring through C‒H···π interaction. The phthalate ring carbon atom C(14) in the molecule at (*x*, *y*, *z*) is in contact with the centroid of the pyrazine ring in the molecule at (−1/2 + *x*, 3/2 − *y*, 1 − *z*); thus interconnects the parallel chain and leads the one-dimensional chains into a two-dimensional layered assembly in **2** (Figure 4d). 

### 3.2. Thermal Analysis

Thermal stability of compounds **1** and **2** were studied by thermogravimetric analysis (TGA) (Figures S1 and S2). Thermal decomposition patterns of both the compounds are complicated and no definite conclusion can be drawn as weight loss at different temperature ranges cannot be matched with the predicted decompositions and consequent loss in weights indicating more than one steps are clubbed together. Compound **1** loses two coordinated water molecules in the temperature range of 137 °C to 190 °C (calcd., 6.93%; found, 11.01%). The weight loss of 34.30% (calcd., 31.77%) in the temperature range of 190 °C to 287 °C may be due to decomposition of one phthalate moiety. Release of one CO_2_ molecule occurs in the range of 287 °C to 372 °C (calcd., 8.47%; found, 12.47%). Decomposition of one bridging ligand occurs in the range of 372 °C to 473 °C with a weight loss of 21.18% (calcd., 18.31%). Complete decomposition of the compound **1** occurs at around 700 °C. For compound **2**, loss of one water of crystallisation takes place in the range 91 °C to 159 °C (calcd., 3.43%; found, 6.29%) and the coordinated water releases in the range of 159 °C to 180 °C (calcd., 3.43%; found, 6.14%). The weight loss of 59.33% (calcd., 62.86%) in the range of 180 °C to 303 °C occurs due to release of two phthalate ligands. From the graph, it can be said that CoO (calcd., 14.42%; found, 9.19%), and CuO (calcd., 15.15%; found, 17.79%) may be the residual species for compounds **1** and **2** respectively. Compound **1** shows continuous decomposition up to 700 °C whereas compound **2** is unstable at higher temperature range (>303 °C).

### 3.3. Theoretical Study

This section is devoted to the theoretical study of the N–H···π interactions observed in the solid state of both the compounds **1** and **2** that have a prominent role in the formation of two-dimensional layers, as explained above. The N–H···π distances are very short in both compounds (see Table 2) thus anticipating that they are energetically relevant. We have focused our attention to the analysis of the influence of the metal coordination on both the donor and acceptor aromatic moieties by using theoretical models of the polymeric chains.

**Figure 5:**
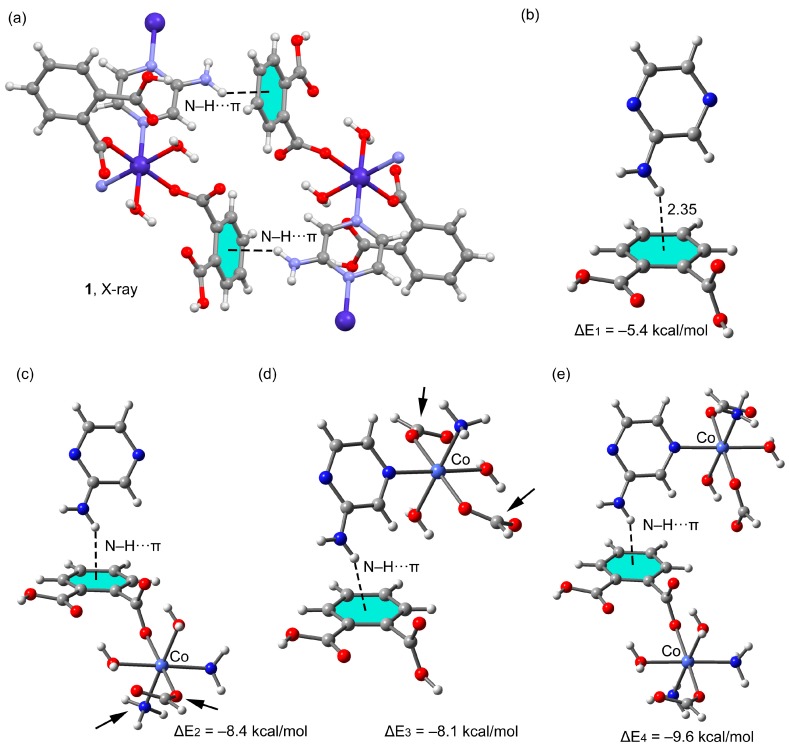
(**a**) X-ray fragment of compound **1**. (**b**–**e**) Theoretical models used to estimate the effect of the metal coordination on the N‒H···π interactions. Distance in Å. The amino group that is interacting with the aromatic ring (50% occupancy) has been considered for this study.

In Figure 5a, we show a fragment of the X-ray structure of compound **1** where the self-assembly of the one-dimensional coordination polymers is highlighted. The antiparallel one-dimensional polymeric chains are connected via the self-complementary N–H···π interactions. Two important factors may influence the strength of the interaction and explain the short distance (2.34 Å). On one hand, the anionic nature of the phthalate ligand that enhances the ability of the π-system as electron donor and, on the other hand, the acidity of the NH group is enhanced by the coordination of the pyrazine to the Co(II) metal center. To verify this hypothesis, we have carried out DFT calculations (BP86-D3/def2-TZVP level of theory) in some model systems and the results are gathered in Figure 5b–e. 

We have first computed the interaction energy using the uncoordinated 2-aminopyrazine ligand and the neutral phthalic acid (see Figure 5b). As a result, the interaction energy is modest (ΔE_1_ = −5.4 kcal/mol). In the second model, we have analyzed the effect of the deprotonation and coordination of the phthalate to the Co(II) using the coordination mode of the crystal structure. In this particular model, one ammonia and one formate ligand (see small arrows in Figure 5c) are used to replace the phthalate and pyrazine ligands. As a result, the interaction energy increases to ΔE_2_ = −8.4 kcal/mol, thus confirming the reinforcement of the N–H···π interaction due to the coordination of the phthalate ligand to the metal center. In a third model, we have studied the influence of the coordination of the 2-aminopyrazine on the interaction energy. In this model, we have used two formate and one ammonia ligands to complete the coordination sphere of the Co(II) metal center to emulate its coordination in the crystal structure (see Figure 5d). The interaction energy of this theoretical model is ΔE_3_ = −8.1 kcal/mol, thus indicating a reinforcement of the interaction with respect to the uncoordinated model. This result also reveals that the coordination of the phthalic acid to the Co(II) metal center has a stronger effect on the interaction than the mono-coordination of the pyrazine ring. Since in the X-ray structure, the pyrazine is di-coordinated, it is expected to have stronger influence on the strength of the interaction. Finally, in the model shown in Figure 5e, we have examined the effect of the coordination in both the pyrazine and phthalic acid ligands. In this case, the interaction energy is ΔE_4_ = −9.6 kcal/mol is considerably more favorable than ΔE_1_ = −5.4 kcal/mol explaining the short experimental distance and key role of this interaction in the solid-state architecture of compound **1**. 

**Figure 6:**
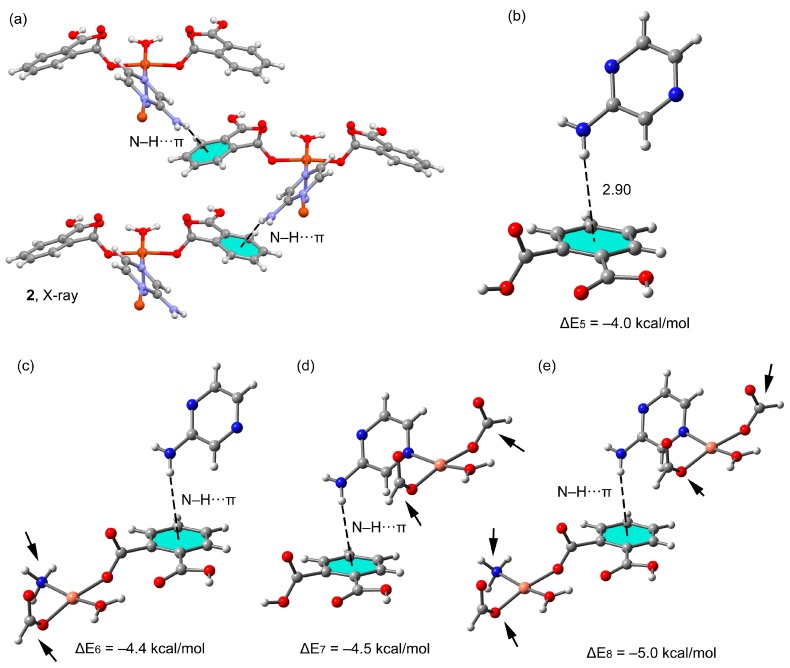
(**a**) X-ray fragment of compound **2**. (**b**–**e**) Theoretical models used to estimate the effect of the metal coordination on the N‒H···π interactions. Distance in Å. The amino group from 2-aminopyrazine moiety that have higher occupancy factor has been taken into account for calculations and to draw the figures.

In Figure 6a, we show a fragment of the X-ray structure of compound **2** where the N–H···π interactions that interconnect the one-dimensional coordination polymers are highlighted. For this compound, we have also initiated the study by computing the interaction energy using the uncoordinated 2-aminopyrazine ligand and the neutral phthalic acid (see Figure 6b). The resulting interaction energy is smaller (ΔE_5_ = −4.0 kcal/mol) than that computed for the same model of compound **1** in agreement with the longer distance observed in **2** (2.89 Å). In the second model, we have studied the effect of the deprotonation and coordination of the phthalate to the Cu(II) using a square planar coordination mode. The axial coordination on the X-ray structure is significantly longer than the rest and, consequently, the elimination of the axial ligand in the theoretical system is a convenient way to convert the polymeric chain to a monomeric one. In this particular model, one ammonia and one formate ligand (see small arrows in Figure 6c) are used to replace the phthalate and pyrazine ligands. As a result, the interaction energy slightly increases to ΔE_6_ = −4.4 kcal/mol, thus confirming the reinforcement of the N–H···π interaction due to the coordination. However, the effect of Cu(II) is weaker than that of Co(II) enhancing the interaction. In the next model, we have studied the influence of the coordination of the aminopyrazine on the interaction energy. In this model, we have used two formate ligands to complete the coordination sphere of the Cu(II) metal center (see Figure 6d). The interaction energy of this theoretical model is ΔE_7_ = −4.5 kcal/mol, thus revealing that the effect of the coordination on the interaction energy is also smaller compared to compound **1**. Finally, in the model shown in Figure 6e, the effect of the coordination in both rings are analyzed. In this case the interaction energy is ΔE_8_ = −5.0 kcal/mol that is approximately the sum of the individual effects (coordination of Cu to phthalic acid enhances 0.4 kcal/mol and the coordination of pyrazine enhances 0.5 kcal/mol).

**Figure 7:**
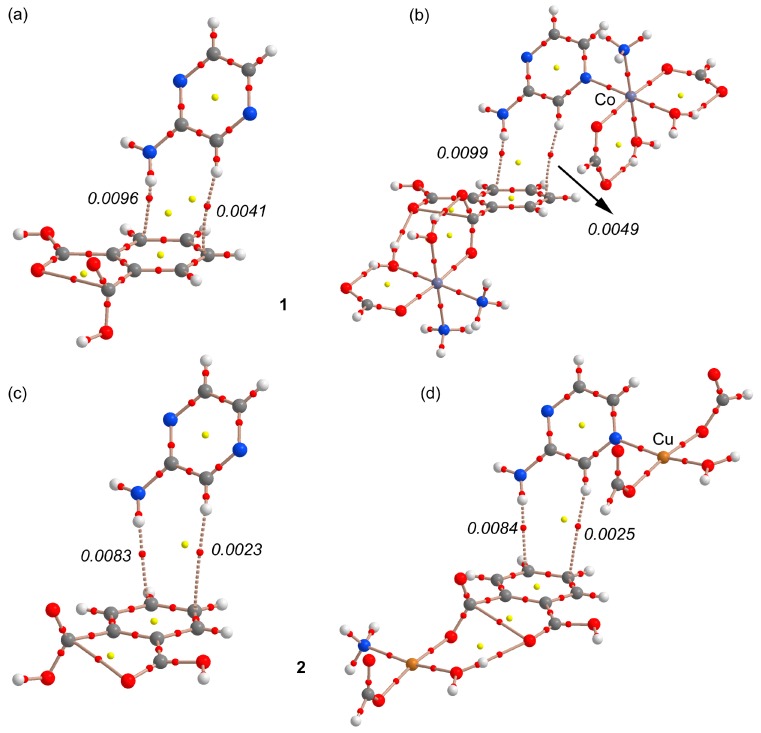
AIM analysis of two compounds **1** (**a**,**b**) and **2** (**c**,**d**). Red and yellow spheres represent bond and ring critical points, respectively. The bond paths connecting bond critical points are also represented by dashed lines. The values of ρ(r) at the bond CP are given in atomic units (a.u.).

We have also carried out the “atoms-in-molecules” (AIM) analysis of several models of compounds **1** and **2**. The presence of a bond path combined with a bond CP (critical point) inter-connecting two atoms is a strong confirmation of interaction [34]. AIM analysis has been recently used to rationalize slight H-bonding differences in polymeric polymorphs [35]. Figure 7 depicts the AIM analysis of the “naked” N–H···π model compounds of **1** and **2** (Figure 7a,c, respectively) and these models where the aromatic ligands are coordinated to their respective metal centers. The distribution of critical points shows that the N–H···π interaction (in all models) is characterized by the presence of a bond CP and bond path connecting the N–H group to one of the carbon atoms of the ring. Moreover, the AIM analysis reveals the presence of an ancillary C–H···π interaction involving one aromatic H-atom. This interaction is also characterized by the presence of one CP and bond path interconnecting the aromatic ring and the aromatic H-atom. Finally, the interaction is further characterized by the presence of a ring CP (yellow sphere) that emerges as a consequence of the formation of a supramolecular ring. 

Since the charge density ρ(r) determined at the bond CP is a good measure of the strength of the interaction, the comparison of the ρ(r) values in the coordinated and uncoordinated compounds provides further insight into the influence of the metal coordination on the binding energy. The ρ(r) values are given in italics in Figure 7. The inspection of the results indicates that the values of ρ(r) are larger in compound **1** than in compound **2**, in good agreement with the energetic analysis commented above and confirming that the value of ρ(r) is good indicator of the interaction strength. Furthermore, the value at the bond CP that characterizes the N–H···π is larger than that at the C–H···π bond CP, suggesting that the N–H···π interaction is stronger, in line with its shorter distance to the ring center. Moreover, the values of ρ(r) at the bond CPs are slightly larger in the compound where the ligands are coordinated to the metal centers, confirming that the coordination strengthens the N–H···π and C–H···π interactions. Moreover, the AIM analysis also reveals that the C–H···π interaction is more reinforced than the N–H···π, likely due to the close proximity to the N-atom of pyrazine that coordinates to the metal center.

**Figure 8:**
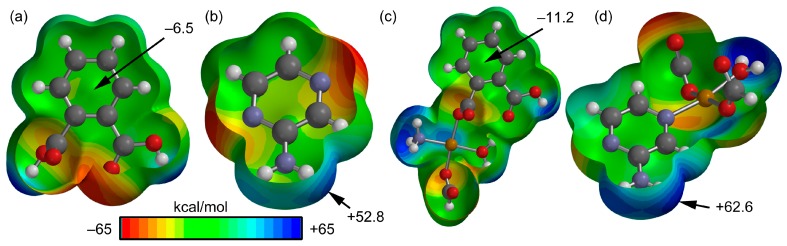
Molecular electrostatic potential (MEP) surfaces computed using the 0.002 a.u. isosurface for (**a**) phthalic acid, (**b**) aminopyrazine, (**c**) phthalate coordinated to Cu(II) and (**d**) aminopyrazine coordinated to Cu(II). The values of MEP at selected points of the surfaces are given in kcal/mol. The level of theory is B3LYP/6-31+G* and the software used to represent the surfaces is Spartan’10 version 1.1.

Finally, to give support to the influence of the metal on the electronic properties of both the N–H acidity and the π-basicity of the aromatic ring, we have computed the molecular electrostatic potential (MEP) surfaces of the models used above for compound **2** (see Figure 6) as an exemplifying case study. The MEP surfaces of all models are shown in Figure 8. It can be observed that the MEP value at π-system of phthalic acid (over the center of the ring) is −6.5 kcal/mol and becomes more negative in the phthalate coordinated to Cu(II), thus indicating that the π-basicity of the ring increases, thus explaining the strongest interaction energy of the model shown in Figure 6c with respect to the uncoordinated model (Figure 6a). Interestingly, the MEP value at the N–H group increases considerably [from +52.8 kcal/mol (Figure 8b) to +62.6 kcal/mol (Figure 8d)], thus revealing that the acidity of the proton increases upon complexation of pyrazine to the Cu(II) metal center, thus increasing its ability to participate in N–H···π interactions.

## 4. Conclusions

Understanding the structural properties of coordination polymers may contribute to achieve the desired rational design of new materials with more predictable solid-state structure. We have synthesized and X-ray characterized two new one-dimensional polymeric compounds of Co(II) and Cu(II) metal centers with 2-aminopyrazine ligand and phthalic acid. Both the compounds exhibit remarkable N–H···π interactions in their crystal structure. The analysis of energies associated to the interactions, including the estimation of influence of the metal coordination has been conducted with the use of DFT calculations. Those results evidence the influence of the metal coordination (specially Co) on the interaction energy that is additionally corroborated with the Bader’s theory of “atoms in molecules”. The mechanism of the synergetic effect has been explained by using molecular electrostatic potential surface calculations revealing its electrostatic nature. 

## Figures and Tables

**Table 1:** Crystal data and structure refinement parameters for **1** and **2**.

Structure	1	2
Empirical formula	C_20_H_18_CoN_3_O_10_	C_20_H_19_CuN_3_O_10_
Formula Weight	519.30	524.92
Temperature (K)	100(2)	293(2)
Wavelength (Å)	0.71073	0.71073
Crystal system	Monoclinic	Orthorhombic
space group	C2/c	Pbca
*a*, *b*, *c* (Å)	17.3919(8), 11.7007(5), 10.3379(5)	11.1679(4), 10.7968(4), 35.0988(11)
α, β, γ (°)	90, 93.5973(14), 90	90, 90, 90
Volume (Å^3^)	2099.59(17)	4232.1(3)
Z/Density (calcd.) (Mg/m^3^)	4/1.643	8/1.648
Absorption coefficient (mm^−1^)	0.883	1.097
F(000)	1064	2152
Crystal size (mm^3^)	0.15 × 0.11 × 0.06	0.16 × 0.10 × 0.07
Limiting indices	−16 ≤ h ≤ 25	−13 ≤ h ≤ 13
	−16 ≤ k ≤ 16	−12 ≤ k ≤ 12
	−14 ≤ l ≤ 15	−41 ≤ l ≤ 41
Reflections collected/unique	12747/3496 [R(int) = 0.0293]	56686/3700 [R(int) = 0.1005]
Completeness to θ (%)	99.7	99.4
Absorption correction	Semi-empirical from equivalents	Semi-empirical from equivalents
Max. and min. transmission	0.917 and 0.864	0.93 and 0.87
Refinement method	Full-matrix least-squares on F^2^	Full-matrix least-squares on F^2^
Data/parameters	3496/176	3700/324
Goodness-of-fit on F^2^	1.060	1.110
Final R indices [I > 2σ(I)]	R_1_ = 0.0351, *w*R_2_ = 0.0756	R_1_ = 0.0505, *w*R_2_ = 0.1247
R indices (all data)	R_1_ = 0.0463, *w*R_2_ = 0.0800	R_1_ = 0.0614, *w*R_2_ = 0.1292
Largest diff. peak and hole (e·Å^−3^)	0.478 and −0.312	0.820 and −0.627

R_1_ = ∑||F_o_| − |F_c_||/∑|F_o_|, *w*R_2_ = [∑{(F_o_^2^ − F_c_^2^)^2^}/∑{*w*(F_o_^2^)^2^}]^1/2^ , *w* = 1/{σ^2^(F_o_^2^) + (*a*P)^2^ + *b*P}, where *a* = 0.0263 and *b* = 3.0768 for **1** and *a* = 0.0462 and *b* = 9.1497 for **2**. P = (F_o_^2^ + 2F_c_^2^)/3 for both the structures.

**Table 2:** Geometrical parameters of hydrogen bonds, N–H∙∙∙π and C–H∙∙∙π interaction for the title polymers (Å, °).

D–H∙∙∙A	d(D–H)	d(H∙∙∙A)	d(D∙∙∙A)	D–H∙∙∙A	Symmetry
Compound **1**					
O(5)–H(5O1)∙∙∙O(3)	0.84	1.97	2.779(2)	159	1 − *x*, *y*, 1/2 − *z*
O(5)–H(5O2)∙∙∙O(2)	0.84	1.94	2.676(2)	144	---
O(4)–H(4)∙∙∙O(2)	0.84	1.74	2.579(2)	174	*x*, 1 − *y*, −1/2 + *z*
N(2)–H(2B)∙∙∙Cg(2)		2.34	3.087(3)	142	−1/2 + *x*, 1/2 − *y*, −1/2 + *z*
N(2’)–H(2’B)∙∙∙Cg(2)		2.40	3.169(2)	146	−1/2 + *x*, 1/2 − *y*, −1/2 + *z*
Compound **2**					
N(3)–H(3A)∙∙∙O(1)	0.86	2.14	2.863(7)	142	3/2 − *x*, −1/2 + *y*, *z*
O(4)–H(4)∙∙∙O(2)	0.82	1.80	2.616(4)	170	1/2 − *x*, 1/2 + *y*, *z*
O(8)–H(8)∙∙∙O(6)	0.82	1.81	2.625(4)	177	1/2 − *x*, 1/2 + *y*, *z*
O(9)–H(9A)∙∙∙O(3)	0.94	1.84	2.751(4)	164	---
O(9)–H(9B)∙∙∙O(7)	0.95	1.77	2.716(4)	170	---
O(10)–H(10A)∙∙∙O(7)	0.88	2.59	3.310(6)	139	1/2 + *x*, 3/2 − *y*, 1 − *z*
O(10)–H(10B)∙∙∙O(6)	0.88	2.15	2.846(5)	136	1 − *x*, 1 − *y*, 1 − *z*
O(10)–H(10B)∙∙∙O(2)	0.89	1.92	2.797(5)	166	1 − *x*, 1 − *y*, 1 − *z*
C(3)–H(3)∙∙∙O(4)	0.93	2.53	3.388(5)	154	1 − *x*, −1/2 + *y*, 3/2 − *z*
N(3)–H(3B)∙∙∙Cg(2)		2.89	3.720(7)	164	1/2 − *x*, *y*, 3/2 − *z*
C(14)–H(14)∙∙∙Cg(1)		2.97	3.558(4)	122	1/2 + *x*, 3/2 − *y*, 1 − *z*
N(3’)–H(3’B)∙∙∙Cg(3)		2.28	2.979(2)	139	1 − *x*, 1 − *y*, 1 − *z*

For polymer **1**, Cg(2) is the centroid of the ring (C2–C7) and for polymer **2**, Cg(1), Cg(2) and Cg(3) are the centroids of (N1/N2/C17–C20), (C2–C7), and (C10–C15), respectively.

## Supplementary Materials

The following are available online at http://www.mdpi.com/2073-4360/10/2/182/s1, Figure S1: TG curve of compound **1**, Figure S2: TG curve of compound **2**, Figure S3: Schematic presentation of the network in (110) plane. The green lines represent the bridging pyrazine moiety whereas the pink lines represent the H-bonding interactions between carboxylate moieties of phthalate anion, Table S1: Selected bond lengths (Å) and bond angles (°) around the metal center of polymer (**1**) determined by X-ray diffraction, Table S2: Selected bond lengths (Å) and bond angles (°) around the metal center of polymer (**2**) determined by X-ray diffraction.
