# Supplementary material for: Coordination Polymers Based on Phthalic Acid and Aminopyrazine Ligands: On the Importance of N–H···π Interactions
Source: Polymers (Basel). 2018 Feb 13;10(2):182. doi: 10.3390/polym10020182 (PMC6415139; doi:10.3390/polym10020182)
Supplement: Supplementary file 1 [file polymers-10-00182-s001.pdf]

Supplementary Materials:

Coordination Polymers Based on Phthalic Acid and Aminopyrazine Ligands: On the Importance of N–H···π Interactions

Anowar Hossain [1], Saikat Kumar Seth [2,*], Antonio Bauzá [3], Subrata Mukhopadhyay [1] and Antonio Frontera [3,*]

[1] Department of Chemistry, Jadavpur University, Kolkata 700032, India; anowar.hossain.chem@gmail.com (A.H.); smukhopadhyay@chemistry.jdvu.ac.in (S.M.)
[2] Department of Physics, Jadavpur University, Kolkata 700032, India
[3] Department of Chemistry, Universitat de les Illes Balears, Crta. de Valldemossa km 7.5, 07122 Palma de Mallorca (Baleares), Spain; antonio.bauza@uib.es
* Correspondence: skseth@phys.jdvu.ac.in (S.K.S.); toni.frontera@uib.es (A.F.); Tel.: +34-971-173-498 (A.F.)

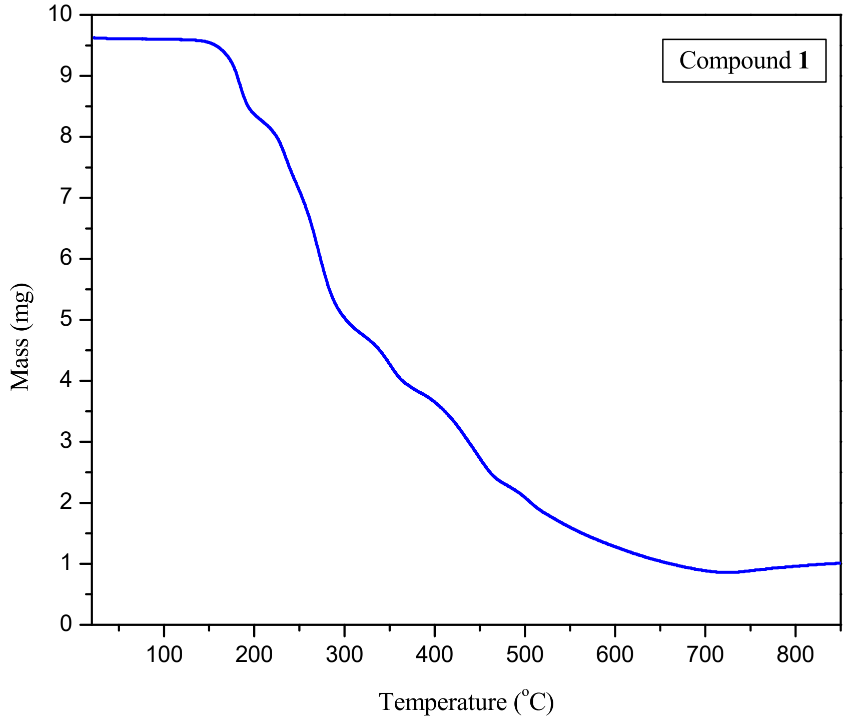

Figure S1. TG curve of compound **1.**

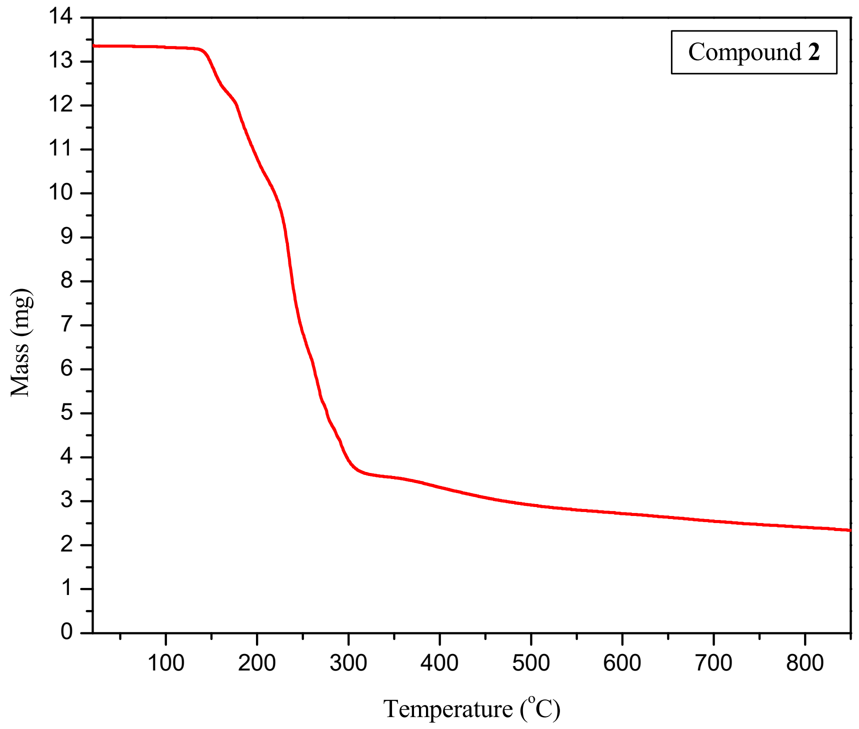

Figure S2. TG curve of compound **2.**

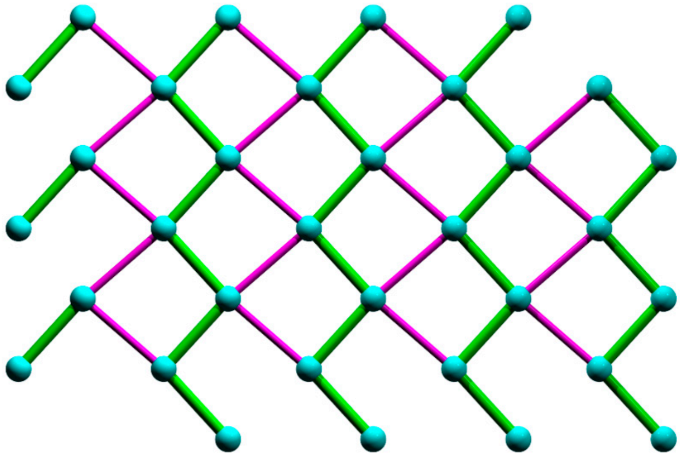

Figure S3. Schematic presentation of the network in (110) plane. The green lines represent the bridging pyrazine moiety whereas the pink lines represent the H-bonding interactions between carboxylate moieties of phthalate anion.

Table S1. Selected bond lengths (Å) and bond angles (°) around the metal center of polymer (1) determined by X-ray diffraction.

Co(1)–O(1)	2.0474(10)	Co(1)–N(1)	2.1705(12)
Co(1)–O(5)	2.1256(11)		
O(1)#1-Co(1)-O(1)	176.33(6)	O(1)-Co(1)-N(1)	90.02(4)
O(1)#1-Co(1)-O(5)	87.62(4)	O(5)-Co(1)-N(1)	178.95(5)
O(1)-Co(1)-O(5)	89.86(4)	O(5)#1-Co(1)-N(1)	87.63(4)
O(1)-Co(1)-O(5)#1	87.62(4)	O(1)#1-Co(1)-N(1)#1	90.02(4)
O(5)-Co(1)-O(5)#1	93.41(6)	O(1)-Co(1)-N(1)#1	92.54(4)
O(1)#1-Co(1)-N(1)	92.54(4)	N(1)-Co(1)-N(1)#1	91.34(7)

Symmetry transformations used to generate equivalent atoms: #1 (-x+1, y, -z+1/2).

Table S2. Selected bond lengths (Å) and bond angles (°) around the metal center of polymer (2) determined by X-ray diffraction.

Cu(1)-O(5)	1.933(3)	Cu(1)-O(9)	1.964(3)
Cu(1)-O(1)	1.950(3)	Cu(1)-N(1)	2.025(3)
O(5)-Cu(1)-O(1)	170.60(12)	O(5)-Cu(1)-N(1)	92.58(11)
O(5)-Cu(1)-O(9)	88.28(11)	O(1)-Cu(1)-N(1)	92.42(12)
O(1)-Cu(1)-O(9)	88.37(12)	O(9)-Cu(1)-N(1)	168.77(16)
